# Strategies for Modulating Oxidative Stress under Diverse Physiological and Pathological Conditions

**DOI:** 10.1155/2018/3987941

**Published:** 2018-12-30

**Authors:** Karolina Szewczyk-Golec, Jolanta Czuczejko, Przemko Tylzanowski, Joanna Lecka

**Affiliations:** ^1^Department of Medical Biology and Biochemistry, L. Rydygier Collegium Medicum in Bydgoszcz, Nicolaus Copernicus University in Torun, Bydgoszcz, Poland; ^2^Department of Nuclear Medicine, Oncology Centre Prof. Franciszek Łukaszczyk Memorial, Bydgoszcz, Poland; ^3^Laboratory for Developmental and Stem Cell Biology, Skeletal Biology and Engineering Research Center, Department of Development and Regeneration, KU Leuven, Leuven, Belgium; ^4^Department of Biochemistry and Molecular Biology, Medical University of Lublin, Lublin, Poland; ^5^Eau Terre Environnement Research Centre, Institut National de la Recherche Scientifique (INRS-ETE), Université du Québec, Québec, Canada

Numerous physiological and pathological conditions are related to the augmentation of oxidative stress. The weakened antioxidant defense and the increased production of reactive oxygen and nitrogen species are thought to be involved in the etiology of cardiovascular, neurodegenerative, and immune diseases as well as cancer, diabetes mellitus, obesity, aging, and others. Obesity, chronic diseases, and age-related disorders such as dementia and Parkinson's disease belong to the major healthcare problems worldwide. Many studies have been conducted to understand the role of oxidative stress in the pathogenesis of chronic diseases. However, the mechanisms and molecular effects of strategies modifying oxidative stress on these conditions are still not well recognized. These strategies include different procedures, such as antioxidant substance supplementation, physical activity, exercise, or therapeutic hypo- and hyperthermia. The expanding of the knowledge in the field of therapies regulating pro/antioxidant balance in the organism seems to be crucial for world healthcare. It is very important to understand how these strategies influence antioxidant processes and protect cells and organs against the deleterious action of reactive oxygen and nitrogen species. Therefore, this special issue focuses on the following aims: (i) to identify new therapies modifying pro/antioxidant balance in health and disease progression and (ii) to understand the biochemical and molecular mechanisms involved in the therapeutic strategies that may decrease oxidative stress and in consequence may influence the inflammatory state.

The selection of original research and review articles in this special issue covers a wide range of topics, including new antioxidant strategies used in diabetes mellitus, arthritis, and nervous system injury as well as in cardiovascular, renal, and oral cavity diseases, oxidative stress in semen with the correlation to male fertility, new biomarkers of aging, and age-related frailty. Additionally, topics of metabolic surgery, exercise, and cryotherapy as the procedures improving pro/antioxidant balance in health and disease are addressed. Moreover, the effect of regular training on the exercise-induced oxidative stress is also discussed.

In the original research article, A. Ahangarpour et al. considered the role of oxidative stress in the pathogenesis of type 2 diabetes mellitus. They observed increased lipid peroxidation, measured as malondialdehyde (MDA) level, and decreased catalase (CAT) activity in the pancreas of diabetic mice, as well as decreased superoxide dismutase (SOD) activity in skeletal myotube cell lines. They also evaluated the antioxidant and antidiabetic effect of plant-derived antioxidant—myricitrin—in the *in vivo* (mice) and *in vitro* (skeletal myotube cell line) studies. Authors observed that the administration of solid lipid nanoparticles (SLNs) of myricitrin enhanced antioxidant capacity and reduced lipid peroxidation in the pancreas and this effect is stronger *in vitro*. Moreover, they discovered that SLN myricitrin administration in diabetic mice protects the muscle and pancreatic tissue from weight loss and damage. In conclusion, the authors claimed that SLN myricitrin shows not only antioxidant but also antidiabetic effect through the improvement of hyperglycemia, hyperinsulinemia, and *β*-cell function index.

The association between increased oxidative stress and type 2 diabetes mellitus (T2D) is also the subject of O. Lushchak et al.'s review. They report that, in patients with T2D, abnormal reactive oxygen species (ROS) concentrations may damage lipid, protein, and nucleic acid molecules, including disturbances in the patterns of gene expression and signal transduction, and also may influence on telomere attrition in pancreatic beta-cells and adipocytes. An increase in ROS levels can then result in the increase of advanced glycation end product (AGE) formation and impair antioxidant defense. The authors emphasized that modulating the processes of ROS, AGE formation, and telomere attrition by innovative technology of metallic nanoparticles (NPs) could provide a promising way for preventing the progression of T2D. Metallic NPs, including cerium oxide, iron oxide, cobalt oxide, copper oxide, manganese dioxide, vanadium pentoxide, gold, silver, and platinum, are known as “nanoantioxidants” and may imitate SOD, CAT, and oxidase and peroxidase activities. O. Lushchak et al. underlined that, due to their properties, NPs have a great clinical potential.

The implication of ROS in diabetic retinopathy (DR) pathogenesis in hyperglycemic conditions is the subject of M.-Y. Wu et al.'s review. According to the authors, excessive glucose oxidation and accumulation of ROS in the retina may lead to local inflammation and endothelial cell death. Endothelial cell death may result in the inflammatory state and microvascular dysfunction in the retina, which further causes blindness. They specified five metabolic pathways, namely, the pathway of sorbitol-aldose reductase, hexosamine, angiotensin II, AGE synthesis, and protein kinase C (PKC), which can be overactivated in hyperglycemic conditions and then become the source of ROS. The authors emphasized that increased oxidative stress damaged the structure and function of the mitochondria leading to the disturbances in levels and activities of mitochondrial SOD, CAT, glutathione peroxidase (GPx), MDA, uncoupling proteins, aldose reductase, AGEs, nitrotyrosine, and 8-hydroxyguanosine. They mentioned decreases in MnSOD expression in DR patients with retinal neuron apoptosis. M.-Y. Wu et al. emphasized that the pathogenesis of DR is complex and has not been completely elucidated; nevertheless, the observation that neurovascular dysfunction may be the result of ROS overproduction is a very interesting issue.

In the research article, Y. Zhang et al. investigated the role of nicotinamide adenine dinucleotide phosphate (NADPH) oxidases (NOXs) in calpain activity, endoplasmic stress (ER), autophagy, and apoptosis during metabolic stress in human cell line ARPE-19, which has functional and structural characteristics similar to retinal pigment epithelial (RPE) cells. Located in the retina, RPE cells are highly metabolically active and play a vital role in maintaining normal visual function. They are vulnerable to oxidative stress and their ROS-induced damage is considered to be involved in the pathogenesis of various ocular diseases. The authors found that Earle's balanced salt solution (EBSS), used as an inducer of cell metabolic stress, resulted in an increase in NOX2, NOX4, p22phox protein, and NOX5 compared to NOX1 in the ARPE-19 cells. Moreover, they also observed reduced ER and autophagy, decreased ROS generation, and alleviated cell apoptosis after suppression of NOXs. The silencing of NOX4, NOX5, and p22phox, but not NOX1, results in the decrease of cell damage. The effect of taurine administration on the cell response to EBSS stress is also investigated. It is demonstrated that taurine attenuated ER, autophagy, and apoptosis in the ARPE-19 cells via the suppression of NOX-derived ROS-mediated calpain induction pathway.

A. B. Sá-Nakanishi et al. studied the actions of fatty acid-derived cyclopentanone—methyl jasmonate (MeJA)—on systemic inflammation and oxidative status in rats with adjuvant-induced arthritis. Authors observed intensified inflammation and increased oxidative stress in arthritic rats. Oxidative stress is demonstrated by increased plasma levels of protein carbonyl groups and higher levels of ROS, lipoperoxides, and carbonyl groups in the arthritic liver. Deficiency in CAT and a very low reduced/oxidized glutathione ratio (GSH/GSSG) are observed in the arthritic liver. Authors claim that impaired ROS scavenging system and the increased production of ROS in arthritic rats are the result of proinflammatory cytokine action. They showed that MeJA administration decreases inflammatory processes, reduces ROS level in the liver, and restores GSH/GSSG ratio but stimulated mitochondrial ROS production in the arthritic rats. The authors prove that this stimulation does not increase hepatic oxidative stress because the effect of MeJA as a ROS scavenger predominates its actions as a stimulator of ROS production.

In turn, X. Li et al. investigated the neuroprotective effect and mechanisms of propofol (anesthetic drug) action in hippocampal neurons expose to ischemia-reperfusion (IR) injury. The results show that treatment of propofol significantly suppresses the apoptosis of hypoxia-reoxygenated hippocampal neurons via the reduction of intracellular calcium overload. The authors hypothesized that propofol may regulate the activation of phosphokinases and phosphatases and thus it would exert neuroprotective effect in IR injury by activating a transcriptional coactivator—Yes-associated protein (YAP). In the study, it is proved that propofol could dephosphorylate YAP, resulting in its activation. This finding supports the therapeutic role of propofol against IR injury in the nervous system.

The aim of C. Tomas-Sanchez et al.'s study is to evaluate the efficacy of prophylactic zinc and therapeutic selenium supplementation against transient hypoxic-ischemic event in cerebral hypoxia-ischemia rat model. The authors reported interesting information about zinc and selenium action against hypoxic-ischemic injury. They claimed that zinc causes a decrease in interleukin-1 (IL-1) and interleukin 23 (IL-23) expression, as well as in oxidative stress intensity (measured as the Cu-Zn-SOD activity), and an increase in chemokine and growth factor levels. In turn, the selenium treatment decreases oxidative stress via both the mechanism of inhibition of inducible nitric oxide synthase (iNOS) and cyclooxygenase-2 (COX-2) expression and via the action of selenoproteins, namely, GPx and thioredoxin reductase, that catalyze peroxide reduction. For this reason, C. Tomas-Sanchez et al. administrated zinc (0.2 mg/kg of body weight daily, i.p.) for 14 days before and after a 10 min common carotid artery occlusion (CCAO) and sodium selenite (6 *μ*g/kg of body weight daily, i.p.) after CCAO for 7 days. The authors found that prophylactic administration of zinc decreases nitrosative/oxidative stress and increases GPx and SOD expression and activity, as well as nitric oxide synthase (eNOS) expression in the temporoparietal cerebral cortex of the examined rats. The therapeutic administration of selenium maintains the effect of zinc up to the late phase of hypoxia-ischemia. Additionally, long-term memory is improved. The results show that the prophylactic zinc and therapeutic selenium administration induces effective neuroprotection in the early and late phases after CCAO.

The cardiovascular disease issue is addressed by J. Zhang et al. who investigated the mechanisms of the potential protective role of exogenous hydrogen sulphide (H_2_S), a gasotransmitter with a variety of cardiovascular protective effects, against myocardial hypertrophy. They found that NaHS significantly reduces the cardiac index of isoproterenol- (ISO-) induced mice, decreases the cross-sectional area of cardiomyocytes, and inhibits the expressions of atrial natriuretic peptide (ANP) and brain natriuretic peptide (BNP) mRNAs. The activities of total antioxidant capacity (T-AOC) and SOD in the myocardium are increased, whereas the level of MDA is decreased and superoxide anion production was attenuated. The expression of optic atrophy 1 (OPA1), a protein important for proper mitochondrial function, is upregulated, while dynamin-related protein 1 (DRP1) expression is downregulated. However, all the above protective effects are unavailable in ISO-induced sirtuin 3 (SIRT3) knockout mice. In summary, exogenous H_2_S supplementation results in the inhibition of ISO-induced cardiac hypertrophy depending on SIRT3, which might be associated with oxidative stress. The authors highlighted that SIRT3 may be a novel therapeutic target for the protective effect of H_2_S against myocardial hypertrophy.

B. Qin et al. investigated the effect of losartan on the prevention and treatment of hyperoxaluria nephrolithiasis. They hypothesized that the overproduction of ROS is associated with renal tubular cell injury in states of high levels of oxalate and calcium oxalate (CaOx) crystals. ROS are involved in the formation of CaOx stones by regulating multiple signaling pathways, including nuclear factor kappa-light-chain-enhancer of activated B cells (NF-*κ*B) and mitogen-activated protein kinase. Authors claimed that NADPH oxidase and angiotensin II (Ang II) are responsible for the production of excessive ROS amounts in hyperoxaluric conditions. They observed that serum Ang II concentration is increased in hyperoxaluric rats. Moreover, the activity of NADPH oxidase is increased, and ROS production and lipid peroxidation levels were enhanced. The authors noticed that losartan reduces renal crystallization via inhibiting NADPH oxidase and decrease of oxidative stress. Hence, losartan may be a potential preventive and therapeutic candidate for hyperoxaluria nephrolithiasis.

Renoprotective effect of platelet-rich plasma (PRP) is the subject of N. Salem et al.'s research. Taking into account that PRP is considered as the source of growth factors that may induce tissue repair, the authors aim to find its application against cisplatin- (CP-) evoked nephrotoxicity in male rats. They found that treatment with PRP reduces creatinine and blood urea nitrogen (BUN), intercellular adhesion molecule-1 (ICAM-1), kidney injury molecule-1 (KIM-1), caspase-3, transforming growth factor (TGF-*β*1) levels, and N-acetyl glucosaminidase (NAG) activity but increases epidermal growth factor (EGF) concentration. Moreover, histopathological investigation reveals the restoration of normal renal tissue architecture after the PRP treatment. The results suggest that PRP may be considered as a promising agent to improve the therapeutic index of cisplatin.

Next, biological activities and potential oral applications of N-acetylcysteine (NAC) is the subject of Y. Pei et al.'s review. The oral cavity is a source of oxidative stress and inflammation induced by environmental insults. Additionally, some dental materials (including resin) have the potential to induce oxidative stress, DNA damage, and inflammatory reactions. Taking into account the therapeutic effects of NAC over a wide range of disorders, its anti-inflammatory, antimicrobial, and anticarcinogenic properties, as well as its acting as a direct antioxidant and a GSH precursor, NAC is considered as a therapeutic agent in oral health care. The authors emphasized antioxidant properties of NAC, pointing that the compound reacts rapidly with hydroxyl radical (^·^OH), nitrogen dioxide (^·^NO_2_), and carbon trioxide ion (CO_3_^·−^) and detoxifies ROS produce by leukocytes. Y. Pei et al. state that NAC exerts protective effect against resin monomer-related cytotoxicity due to its antioxidant properties and because it reacts directly with the methacrylic group of resin monomers, reducing the availability of free dental resin monomers. The authors underlined that clinical efficacy of NAC needs further investigations that should especially consider NAC application in dental, implantable, and intracanal materials, the use of NAC alone or with other drugs to treat oral lichen planus. Also, the clinical effectiveness of NAC for the treatment of wound healing and the clinical application of NAC as an anticancer adjuvant for oral cancer treatment should be taken under the account.

In their research, D. Chyra-Jach et al. evaluated the parameters of inflammatory processes (interleukin 12 (IL-12), interleukin 8 (IL-8), monocyte chemoattractant protein-1 (MCP-1), and macrophage inflammatory protein-1*β* (MIP-1*β*)), oxidative stress (MDA), and antioxidant defense (albumin, uric acid (UA), total SOD, Cu-Zn-SOD, Mn-SOD, and GPx activities) in patients with abnormalities in spermogram. The level of MDA is significantly higher in seminal plasma and significantly lower in spermatozoa lysate in males with spermogram abnormalities. According to the authors, the levels of MDA, MCP-1, and IL-8 in seminal plasma negatively correlate with sperm motility. Moreover, D. Chyra-Jach et al. showed a decrease in total SOD and Mn-SOD activities but an increase in the activity of spermatozoa lysate Cu-Zn-SOD and an increase in the activity of GPx from seminal plasma in males with spermogram abnormalities. The authors found positive correlation between SOD activity in spermatozoa lysate and sperm volume, sperm cell count, rapid progressive motility after 1 hour, and motile spermatozoa after 24 hours. Their results show the protective role of SOD against oxidative stress in semen. As the authors explained, Mn-SOD may protect the mitochondria against prolonged oxidative stress, which may lead to mitochondrial DNA (mtDNA) damage, mitochondria disruption, ATP pool depletion, and sperm motility impairment. Additionally, seminal plasma and spermatozoa contain nonenzymatic ROS scavengers, such as UA and albumin. The levels of UA and albumin in seminal plasma are significantly higher in males with spermogram abnormalities in the presented research. The authors concluded that abnormalities in spermogram may be related to the decrease in activity of Mn-SOD in spermatozoa and increase in levels of chemokines in seminal plasma.

Relationships between standard semen parameters, markers of oxidative stress, and antioxidant defense functions were also the subject of M. Dobrakowski et al.'s study. Their observations suggested higher activity of enzymatic antioxidant defense (SOD, CAT, and glucose 6-phosphate dehydrogenase (G6PD)) in the group of males with excellent sperm quality (EX) than in the group with mediocre (ME) semen quality. According to the authors, higher sperm motility in EX is associated with intensive metabolism and in a consequence with more intense ROS production than in ME. Comparable ROS levels in both groups indicated that the antioxidant defense system in EX was more effective. Moreover, the authors demonstrated that the levels of cytokines do not differ between the examined groups suggesting that the semen of fertile males is rather homogenous with respect to immune system parameters.

Next, I. Rusanova et al. evaluated microRNAs (miRNAs) as possible biomarkers of age and frailty and their correlation with oxidative and inflammatory level in human blood. The authors analyzed three inflammation-related miRNAs (miR-21, miR-146a, and miR-223), one miRNA related with the control of melatonin synthesis (miR-483), plasma cytokines (IL-6, IL-8, IL-10, and TNF*α*), plasma advanced oxidation protein products (AOPPs), and lipid oxidation products (LPOs) in three groups of patients: healthy (control), aged robust, and aged fragile (with sarcopenia). The aged fragile subjects have higher miR-21, miR-223, miR-483, all cytokines, TNF*α*/IL-10, AOPP, and LPO levels than controls. Increased miR-223, miR-483, all cytokines, AOPP, and LPO levels are observed in aged robust group. Positive correlations between miR-21 and AOPP and between miR-483 and IL-8 are detected. Furthermore, the authors positively correlated the expression of miR-21 and the TNF*α*/IL-10 ratio with the presence of frailty. Their findings confirm that chronic inflammation and oxidative stress accompanied aging and age-related frailty. I. Rusanova et al. underlined that miR-21and TNF*α*/IL-10 ratio may be considered as possible biomarkers for aged-related frailty and that the evaluation of stable miRNAs in the blood gives new possibility in systemic biomarker research.

The influence of exercise training on water-pipe smoke (WPS) exposure-induced increase of airway resistance, lung inflammation, oxidative stress, and DNA damage was a subject of A. Nemmar et al.'s research. This project was carried out on mice exposed to WPS in the period of two months. The authors observed that WPS induces a significant increase in tumor necrosis factor *α* (TNF*α*), interleukin 6 (IL-6), and 8-isoprostane levels in lung homogenates, stimulates the expression of NF-*κ*B, and nonsignificantly influences the expression of nuclear factor 2 (Nrf2). Exercise training significantly reduces the effect of WPS on inflammatory and oxidative stress markers, averts DNA damage, inhibits the effect of NF-*κ*B overactivation, and activates the Nrf2 signaling pathway. The authors emphasized that the protective impact of regular exercise training could be explained by its anti-inflammatory and antioxidant effects.

B. Skrzep-Poloczek et al. examined the influence of duodenal-jejunal omega switch surgery (DJOS) in combination with high-fat (HF) or control (CD) diet on the antioxidant status in the soleus muscle of rats. The authors emphasized that both obesity and chronic use of fat diet are related to enhance oxidative stress, which in turn causes many unfavorable health problems. Among these consequences, altered lipid and glucose metabolism in skeletal muscle may be observed. Under obesogenic conditions, including high-fat diet, the ability of the muscle tissue to oxidize the fat content was strongly reduced and could lead to an increased level of ROS. Surgical treatment of obesity may be considered as a metabolic surgery because it causes not only a reduction in body weight but also influences different metabolic pathways. Thus, in the Skrzep-Poloczek et al. experiment, after eight weeks of HF or CD diet, rats were subjected to DJOS or control (SHAM) surgery. After surgery, half of DJOS/SHAM rats had a changed diet, and half had the same type of food. The authors observed significantly lower CAT and GPx activities in the rat soleus muscle after DJOS, regardless of the type of diet. In turn, the activities of CAT, SOD, glutathione reductase (GR), Cu-Zn-SOD, and GPx are altered in the CD/HF or HF/CD group. After DJOS, the lowest muscle concentration of MDA is observed in the CD/CD group and the highest in the CD/HF group. It was shown in this study that DJOS surgery significantly decreases antioxidant systems in the soleus muscles of rats. CD/HF and HF/CD dietary patterns lead to an increase in antioxidant activity, while remaining on unchanged diet (CD or HF) is associated with reduced oxidative stress. The authors concluded that metabolic surgery together with mixed dietary patterns could be used as a strategy to modulate oxidative stress under pathological conditions.

In their study, A. Stanek et al. aimed to estimate the impact of whole body cryotherapy (WBC) performed in a closed cryochamber on oxidative stress in patients with ankylosing spondylitis (AS). WBC is a method of physical medicine, used in the treatment of rheumatic and inflammatory diseases and muscle spasticity. In the experiment, the effect of ten WBC procedures lasting 3 minutes a day with a subsequent 60-minute session kinesiotherapy on oxidative stress in male AS patients was investigated. The WBC group was compared to the kinesiotherapy (KT) group. To assess the disease activity, the Bath Ankylosing Spondylitis Diseases Activity Index (BASDAI) and Bath Ankylosing Spondylitis Functional Index (BASFI) were calculated. The antioxidant enzymatic and nonenzymatic status, lipid peroxidation products, total oxidative status (TOS), and oxidative stress index (OSI) were measured one day before the beginning and one day after the completion of the research program. According to the authors, WBC procedures performed in a closed cryochamber with subsequent kinesiotherapy significantly decrease oxidative stress as well as BASDAI and BASFI indexes in AS patients during the active phase of the disease.

Finally, Y. Spanidis et al. evaluated the effects of the training background on free radical generation and adaptations after eccentric exercise. In the experiment, trained and untrained volunteers performed eccentric exercise. Biomarkers of oxidative damage and the antioxidant profile of the participants were measured in plasma and erythrocyte lysate at baseline and 24, 48, and 72 hours after the conducted exercise. The authors found more severe oxidative damage, weaker antioxidant status, and weaker radical scavenging activity in the untrained group compared to the trained participants. Their research showed that trained individuals are less susceptible to oxidative damage, suggesting that generalized nutritional recommendations regarding recovery after exercise should be avoided. The authors emphasized that individualized nutritional approach could help to fine-tune the recovery process and consequently improve health status and performance after eccentric exercise.

Overall, the work reported in this special issue highlights the significance of different strategies modulating oxidative stress in diverse physiological and pathological conditions. Moreover, the attention is focused on our understanding regarding the molecular mechanisms of antioxidant action of some interesting procedures, including the use of specific substances, exercise training, cryotherapy, or metabolic surgery. New insight into the effect of regular training on pro/antioxidant balance is also provided.

